# Depth of soil compaction predominantly affects rice yield reduction by reproductive-stage drought at varietal screening sites in Bangladesh, India, and Nepal

**DOI:** 10.1007/s11104-017-3265-2

**Published:** 2017-05-10

**Authors:** Suresh Prasad Singh, Abhinav Jain, M. S. Anantha, Santosh Tripathi, Subarna Sharma, Santosh Kumar, Archana Prasad, Bhawana Sharma, Biswajit Karmakar, Rudra Bhattarai, Sankar Prasad Das, Shravan K. Singh, Vinay Shenoy, R. Chandra Babu, S. Robin, Padmini Swain, J. L. Dwivedi, Ram Baran Yadaw, Nimai P. Mandal, T. Ram, Krishna Kumar Mishra, S. B. Verulkar, Tamal Aditya, Krishna Prasad, Puvvada Perraju, Ram Krishna Mahato, Sheetal Sharma, K. Anitha Raman, Arvind Kumar, Amelia Henry

**Affiliations:** 1 Bihar Agricultural University, Sabour, Bihar India; 2 Barwale Foundation, Himayatnagar, Hyderabad, Telangana India; 3 Central Rainfed Upland Rice Research Station, Jharkand, Hazaribag, India; 4 Nepal Agricultural Research Council Regional Agriculture Research Station, Khajura, Banke, Nepalgunj, Nepal; 5 Indian Council of Agricultural Research, Research Complex for Eastern Region, Patna, Bihar India; 6 Indira Gandhi Agricultural University, Raipur, Chhattisgarh India; 7 Bangladesh Rice Research Institute, Regional Station, Rajshahi, Bangladesh; 8 Regional Agricultural Research Station, Tarahara, Sunsari Nepal; 9 ICAR Research Complex for North Eastern Hill Region, Lembucherra, Tripura India; 10 Banaras Hindu University, Varanasi, Uttar Pradesh India; 11 Tamil Nadu Agricultural University, Coimbatore, Tamil Nadu India; 12 National Rice Research Institute, Cuttack, Odisha India; 13 Crop Research Station, Narendra Dev University of Agriculture and Technology, Masodha, Kumar Ganj, Faizabad, Uttar Pradesh India; 14 National Rice Research Program, Hardinath, Baniniya, Janakpurdham, Nepal; 15 Indian Institute of Rice Research, Rajendranagar, Hyderabad, Telangana India; 16 Birsa Agricultural University, Ranchi, Jharkhand India; 17 Jawaharlal Nehru Krishi Viswa Vidyalaya, Rewa, Madhya Pradesh India; 18 International Rice Research Institute, Los Baños, Laguna Philippines

**Keywords:** Drought, Rice, Soil, Rainfed lowland

## Abstract

**Aims:**

Drought is the major constraint to rainfed rice productivity in South Asia, but few reports provide detailed characterization of the soil properties related to drought stress severity in the region. The aim of the study was to provide a compilation of drought breeding network sites and their respective levels of drought stress, and to relate soil parameters with yield reduction by drought.

**Methods:**

This study characterized levels of drought stress and soil nutrient and physical properties at 18 geographically distributed research station sites involved in rice varietal screening in Bangladesh, India, and Nepal, as well as at farmers’ fields located near the research stations.

**Results:**

Based on soil resistance to penetration profiles, a hardpan was surprisingly absent at about half of the sites characterized. Significant relationships of depth of compaction and yield reduction by drought indicated the effects of soil puddling on susceptibility to cracking, rather than water retention by hardpans, on plant water availability in this region. The main difference between research stations and nearby farmers’ fields was in terms of soil compaction.

**Conclusions:**

These results present an initiative for understanding the range of severities of reproductive-stage drought stress in drought-prone rainfed lowland rice-growing areas in South Asia.

**Electronic supplementary material:**

The online version of this article (doi:10.1007/s11104-017-3265-2) contains supplementary material, which is available to authorized users.

## Introduction

Drought stress has been established as the major constraint to productivity in rainfed lowland rice (Widawsky and O’Toole 1990; Huke and Huke 1997; Pandey and Bhandari 2008), but detailed reports of the types and severities of drought stress affecting rainfed lowland rice crops are limited. Due to the water requirements of the rice plant which peak during reproductive stage (Hsiao and Namuco 1980), estimates of mean rainfall during the rice crop growing season cannot accurately predict yield under drought, and more detailed information on the distribution of rainfall throughout the growing season and water-holding capacity of the soils of rainfed lowland rice-growing areas is needed (Serraj et al. 2009; Haefele et al. 2014). Recent advances in development of drought-tolerant rice varieties have focused on imposing severe reproductive stage stress which has facilitated selection of the most drought tolerant genotypes (Kumar et al. 2008; Kumar et al. 2014), but it is not known if that is the predominant type of drought stress causing yield losses in farmers’ fields. Addressing these knowledge gaps about the types of drought stress occurring in rainfed rice fields and the characterization of drought-prone sites on both global and regional scales will be useful in targeting breeding programs to certain types of drought stress, and for developing crop management strategies specific to drought-tolerant varieties (Haefele et al. 2016).

In this study, we focused on the region in South Asia where it is estimated that drought-prone areas of rainfed lowland rice cover 0.8, 7.3, and 0.27 million ha in Bangladesh, India, and Nepal, respectively (Pandey and Bhandari 2008). This region has also supported a drought breeding network that has been active for decades (Zeigler and Puckridge 1995) and has been a key evaluation system for release of drought tolerant rice varieties (Mandal et al. 2010; Verulkar et al. 2010; Swamy et al. 2013). Remote sensing has recently provided advancements in estimation of the area of drought-prone land in this region in Bangladesh (Mottaleb et al. 2015) and in the state of Odisha, India (Gumma et al. 2015). These remote sensing technologies use spectral reflectance combined with rainfall from satellite data, validated by field-plot data. Simulation modeling predicts that climate change will affect rice yields of South Asia differently depending on the characteristics of each location (Li et al. 2015). These simulation modeling estimates have incorporated site characteristics from global databases. Therefore, detailed direct measurements quantifying the drought stress in terms of soil, the severity of the drought stress, and yield reduction by drought could benefit both the further refinement of remote sensing and simulation modeling technologies to quantify and predict drought stress as well as improve our understanding of these target regions.

Environmental and soil characterization, as well as water balance modeling, of rainfed lowland rice in the Mekong region (Cambodia, Laos, and Thailand; Fukai and Ouk 2012; Inthavong et al. 2012) indicates that typical edaphic constraints to rainfed rice productivity include the presence of a hard pan that impedes root growth, low nutrient availability, and variability in water availability due to topography and low clay content (Cairns et al. 2011; Inthavong et al. 2011; Haefele et al. 2014). In the current study of drought-prone rainfed rice-growing regions of South Asia, we hypothesized that the presence of hardpans and clay content, owing to their direct effect on soil-water dynamics and water-holding capacity, would be the main factors related to yield reduction by drought, in addition to rainfall. We focused on characterizing the soil drought stress progression at a range of research stations across the region in order to provide a compilation of the conditions in which the drought breeding network trials were conducted and to understand which parameters were most closely related to yield reduction by drought across sites.

## Materials and methods

### Experimental sites

**Fig. 1:**
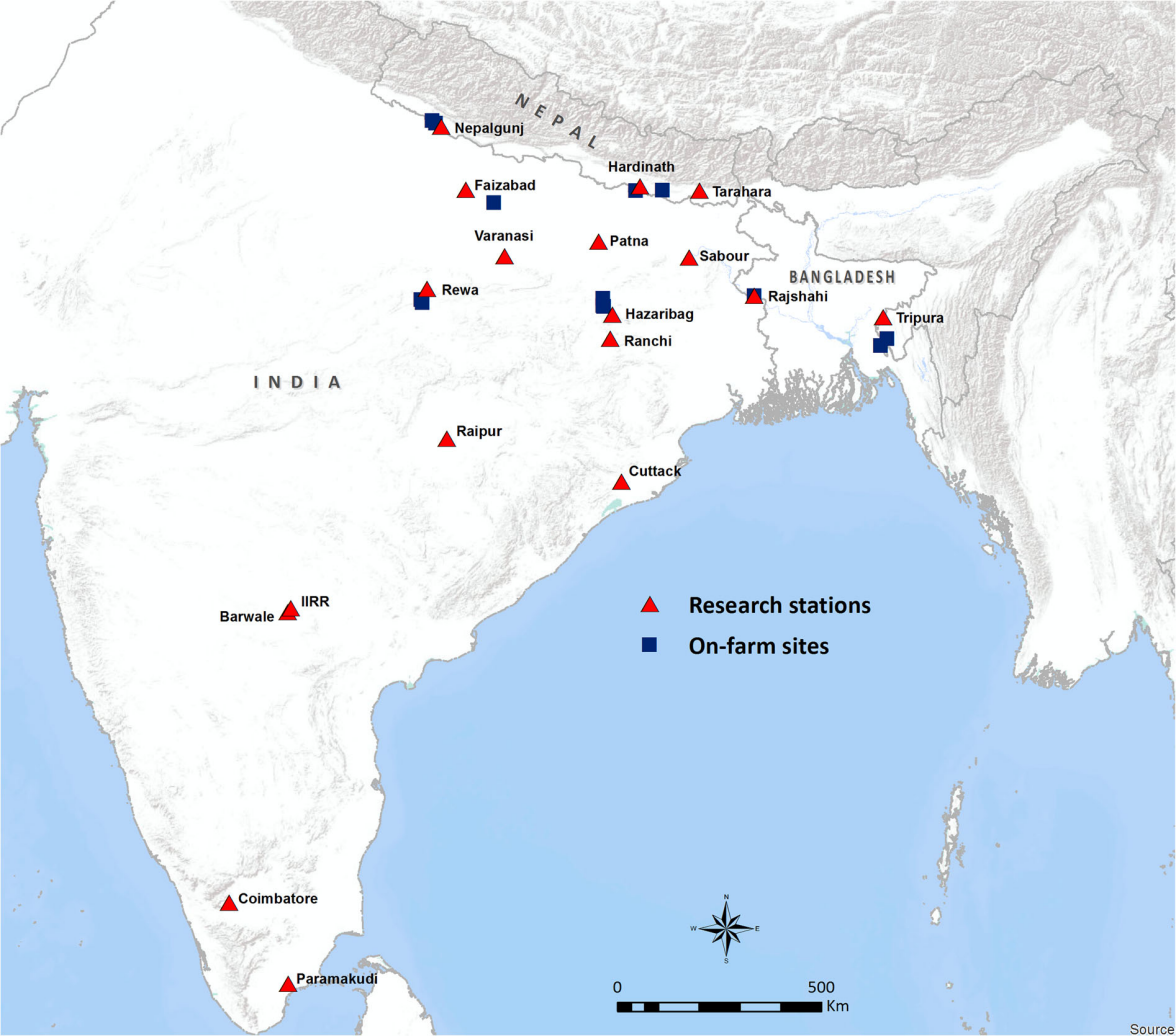
Locations of the rice drought screening network research station and on-farm sites characterized in this study

Drought screening trials were conducted on research stations at eighteen different sites in Bangladesh, India, and Nepal (Table 1, Fig. 1) as part of a research program to identify drought tolerant rice genotypes for each region within a drought-breeding network. The experiments were conducted following a range of land-use histories, especially in terms of the time over which the soil had been cultivated by puddling (Table 1). The sets of rice genotypes being tested were composed of early maturing advanced breeding lines (100–120 days to maturity; see Supp. Table 1 for ranges in time to flowering) and checks with a range of drought tolerance levels at reproductive stage. During the *Aman*/ *Kharif/ Barkha* (wet) season from 2012 to 2014, 32–70 genotypes were tested per season in both irrigated control and reproductive-stage drought stress treatments at the research station sites. Similar sets of genotypes were planted at each location. All trials were arranged in an alpha-lattice design with three replications per genotype in puddled transplanted fields, except at Paramakudi where trials were dry-direct seeded. Plot size ranged from 2.4–3.2 m^2^. Similar recommended agronomic management practices were followed at each location: 21–25 days old seedlings were transplanted at 20 cm × 15–20 cm spacing. Inorganic NPK fertilizer doses ranging up to 90–60–40 kg ha^−1^ were applied, with P and K applied as a single basal dose at transplanting, and N applied in three splits (45 kg ha^−1^ at transplanting and 22.5 kg ha^−1^ at about 28 and 50–56 days after transplanting, depending on timing of rainfall). Weeds and pests were managed as needed.

**Table 1:** Locations of the research station sites, land use history, and the average planting delay of the drought stress treatment compared to the irrigated control treatment

Country	Location (Abbreviated)	Location of experiment	Land use history	Ave. planting delay (d)
Bangladesh	Rajshahi	Rajshahi	mixed puddling and dry seeding previous to puddling for 20+ years	0
India	Barwale	Maharajpet, Ranga Reddy District, Telangana	fallow previous to puddling for 6 years	18
	Coimbatore	Coimbatore, Tamil Nadu	puddled for 100+ years with occasional direct seeding	0
	Cuttack	Cuttack, Odisha	puddled for 20+ years	0
	Faizabad	Masodha, Kumar Ganj, Faizabad, Uttar Pradesh	puddled for 30 years with occasional direct seeding	25
	Hazaribag	Hazaribag, Jharkand	uncultivated previous to puddling for 15 years	5
	IIRR	Patencheru, Telangana	puddled for 50+ years	0
	Paramakudi	Paramakudi, Tamil Nadu	dry plowing (no puddling)	n/a
	Patna	Patna, Bihar	puddled for 7 years	25
	Raipur	Raipur, Chhattisgarh	puddled for 15+ years	20
	Ranchi	Ranchi, Jharkhand	puddled for 30+ years	10
	Rewa	Rewa, Madhya Pradesh	puddled for 10 years	17
	Sabour	Sabour, Bhagalpur, Bihar	puddled for 20+ years with rice-wheat or rice-chickpea rotation	10
	Tripura	Lembucherra, Tripura	uncultivated previous to puddling for 3 years	26
	Varanasi	Varanasi, Uttar Pradesh	puddled for 12 years	14
Nepal	Hardinath	Hardinath, Baniniya, Janakpurdham	puddled for 15 years	20
	Nepalgunj	Khajura, Banke	puddled for 30 years	9
	Tarahara	Tarahara, Sunsari	puddled for 30 years with rice-wheat rotation	0

Additional drought screening trials were conducted at on-farm sites located near the research stations (Fig. 1). The on-farm sites were each managed according to local farmers’ practices and the soils were characterized as part of this study for comparison with conditions at research stations. Land use history at the on-farm site near Hazaribag was reported to be direct-seeding and beushening preceding puddling for the past 20 years, and near Tripura the soil at the on-farm site was estimated to have been puddled for 80 years. The on-farm sites in Nepal were likewise estimated to have been puddled for many years preceding these experiments.

### Management of experimental treatments

Trials at each site were conducted according to a common protocol for water management under natural field conditions, and the environmental conditions were characterized as part of this study to determine the variability among drought screening trials that can occur among sites using the same protocol.

The experimental fields at each research station were selected based on their possible drainage control, leveling, and general uniformity in soil characteristics as recommended for rice drought screening trials (Verulkar et al. 2010). An irrigated control treatment, in which standing water was maintained in the field from transplanting until physiological maturity, was included at each research station trial, at least 10 m away from the drought stress treatment. Both treatments were located on the research station, except in Rajshahi where both treatments were at an on-farm site, and in Tripura where the irrigated control treatment was at an on-farm site in 2014. To implement the drought stress treatments at some sites, the sowing and transplanting were delayed up to 26 days as compared to the irrigated control treatments to increase the likelihood of coinciding crop reproductive stage with the cessation of monsoon rains (Table 1). In each drought stress trial, irrigation was maintained for approximately one month after transplanting and drained, after which the progression of the drought stress treatment varied depending on rainfall and other site characteristics characterized as part of this study.

### Observations of grain yield and soil characteristics

#### Grain yield

Replicated grain yield data per plot of individual entries were recorded separately from each control and stress trial, converted into grain yield per hectare, and averaged separately for the irrigated control and drought stress treatments. The average data obtained in each year from each treatment were used to calculate the percent yield reduction for each site as follows: % yield reduction = (yield under control – yield under stress) / yield under control × 100. This approach of averaging the yield data from all entries per site and normalizing the yield under stress for the yield under irrigated control conditions allowed a focus on environmental characteristics affecting drought response in rice, in contrast to using the absolute yield under drought values which would have also been strongly influenced by other variation among sites affecting general plant growth. The severity of the drought stress treatment in each trial was classified according to the % yield reduction as described by Kumar et al. (2009).

#### Environmental characterization

During the drought stress period, soil moisture in the drought stress treatments was monitored with tensiometers (Soilmoisture Equipment Co) installed at a depth of 30 cm. Water table depth to 1 m was monitored through a perforated PVC tube, and rainfall was recorded at weather stations located nearby the trials. These measurements were collected from 60 to 100 days after sowing (DAS), which approximately represents the reproductive stage of the genotypes evaluated. The number of sites in which tensiometer, water table, and rainfall results were included varied by year depending on each site’s ability to collect the data.

For soil chemical analysis, a composite sample of 5 locations per stress trial field was collected from a depth of 0–15 cm. Soil P, K, pH were analyzed at the Soil, Plant and Water Analysis Laboratory at Sardar Vallabhbhai Patel University of Agriculture and Technology (IRRI-SVPUAT Lab), Modipuram, or at the International Crops Research Institute for the Semi-Arid Tropics (ICRISAT) Soil Chemistry Laboratory. A subset of the same 0–15 cm sample was submitted for particle size analysis by hygrometer method at the IRRI-SVPUAT Lab (Modipuram) or at the ICRISAT Soil Physics Laboratory. Soil type was determined from the particle size results using the ‘soiltexture’ script in R v. 3.3.1 (The R Core Team, 2016). Soil from one location at each research station stress trial field was sampled using a 100 cm^3^ core (Eijkelkamp, Netherlands) at depths of 5–10 cm and 25–30 cm, dried overnight at 102 °C and weighed to determine bulk density. A loose soil sample was taken from 5 to 10 cm and 25–30 cm as a composite of 3 locations per stress trial field from research stations only, and subjected to water retention analysis by packing into 19.63 cm^3^ cores, saturating, equilibrating in pressure plates at 10, 300, 500, and 1500 kPa, and determining the consequent gravimetric water content at the ICRISAT Soil Physics Laboratory.

Soil mechanical resistance to penetration and the presence of a hardpan was determined using a cone penetrometer (CP20, Rimik, Australia) to a depth of 80 cm. The same penetrometer was used during grain filling at all sites. Relative differences in soil moisture at the time of penetrometer measurements were characterized by volumetric soil moisture at the soil surface (0–5 cm: Delta Theta Kit HH2 moisture meter, UK), which ranged from 14.2–46.2% (Supp. Table 2).

#### Statistical analyses

To compare the soil attributes measured at research stations with those measured at on-farm sites, a t-test was performed in R in which 5–6 research station sites were compared with 6–7 farm sites for each parameter. To relate the soil and environmental parameters to yield reduction by drought, a correlation matrix (Supp. Table 3) revealed a high correlation (>0.7 correlation coefficient) of soil exchangeable K^+^ as well as three of the pressures at which soil water retention was measured with other parameters; therefore, those four parameters were removed from the subsequent multiple regression analysis. Step-wise multiple regressions were performed using Statistical Tool for Agriculture Research (STAR) v. 2.0.1 (International Rice Research Institute) to relate yield reduction by drought for each year of the study with the rainfall from 60 to 100 DAS for each year and soil parameters measured at one time point only. To further assess groupings of the soil and environmental parameters measured with yield reduction by drought, a Principal Component Analysis (PCA) was conducted in STAR using Kaiser’s stopping rule and Scree test along with the % of variance to identify the principal components for interpretation and evaluation in a biplot. For simplicity, water retention values from the depth of 5–10 cm only were used in the PCA. From the compiled % yield reduction and environmental characterization data from 16 research-station sites used for the correlation matrix and PCA, 114 out of 902 data points (~13%) were missing due to the logistics of conducting these measurements at many remote locations. In order to include as many sites as possible in the analysis of environmental characteristics related to % yield reduction by drought, the missing data points from 16 sites were imputed by Multivariate Imputation by Chained Equations using the ‘mice’ script in R.

## Results

### Soil characterization

**Fig. 2:**
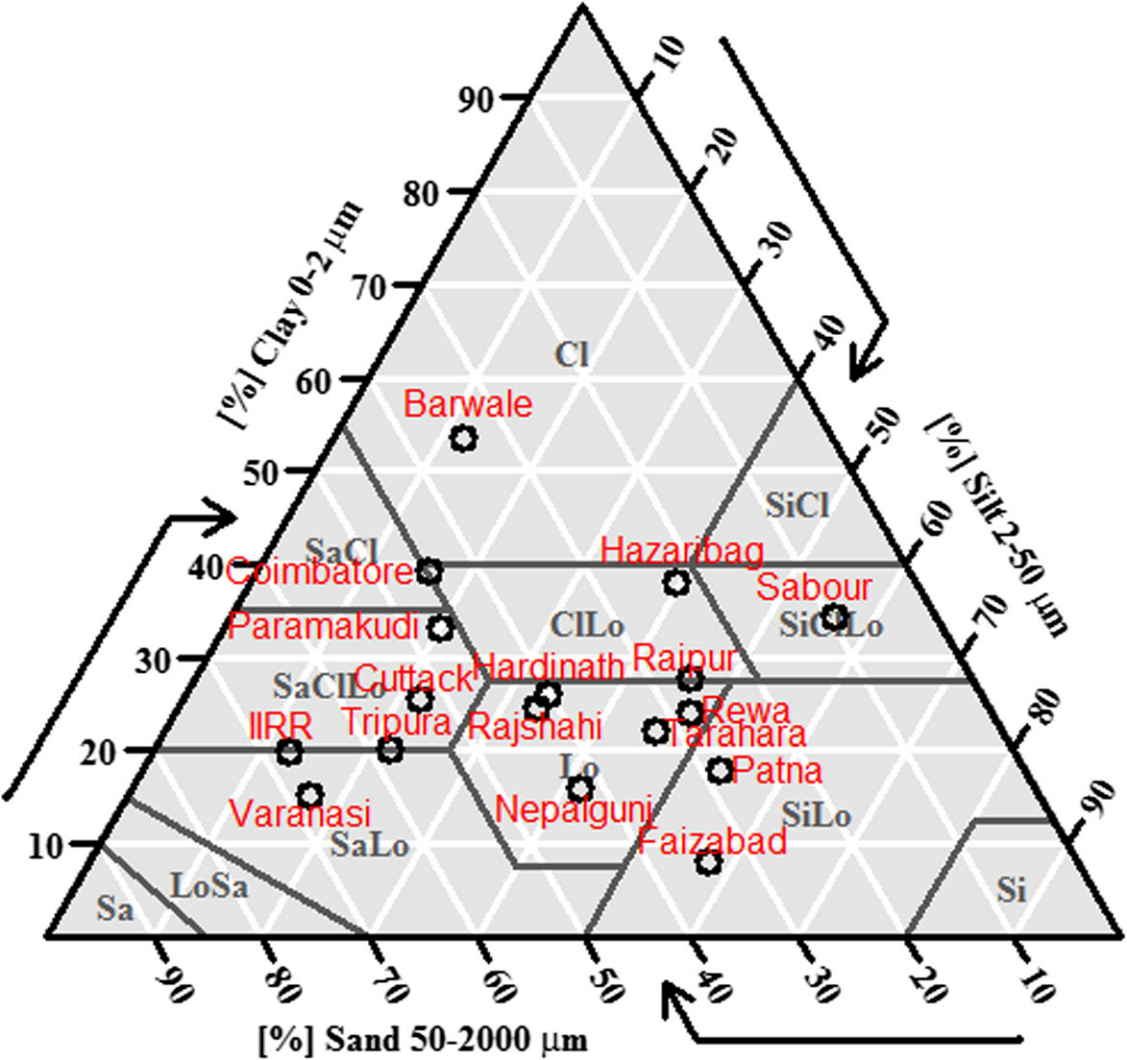
Soil types based on percentages of sand, silt, and clay from the drought screening network research station sites characterized in this study, according to the USDA soil texture triangle

Although each site was selected to represent an environment characteristic of what is considered to be typical rice-growing conditions in drought-prone regions of South Asia, considerable variation in soil characteristics was observed among sites. Soil available P ranged from 3.7 mg kg^−1^ (Tripura) to 70 mg kg^−1^ (IIRR); exchangeable K ranged from 44 mg kg^−1^ (Cuttack) to 584 mg kg^−1^ (Barwale); pH ranged from 4.6 (Tripura) to 8.4 (Varanasi); and bulk density ranged from 1.27 g cm^−3^ (Tarahara, 5–10 cm depth) to 1.88 g cm^−3^ (Hardinath, 25–30 cm depth) (Table 2). About seven different soil texture types were represented among the research station sites including sandy loam, silty loam, and clay (Fig. 2). The % sand and % clay levels were not highly correlated across sites (correlation coefficient = −0.35; Supp. Table 3).

**Table 2:** Soil available P, exchangeable K, and pH from 0 to 15 cm, bulk density (bd) from 5 to 10 cm and 25–30 cm, and the depth of the maximum penetrometer reading at the research station sites characterized in this study (except Rajshahi, where all trials were on-farm)

Location	Avail-P (ppm)	Exch-K (ppm)	pH	bd 5–10 cm (g cm^−3^)	bd 25–30 cm (g cm^−3^)	Depth of max. penetrometer reading (cm)
Barwale	7.9	584	7.9	1.76	1.68	4
Coimbatore	21.6	280	7.9	1.48	1.75	-
Cuttack	24.4	44	5.4	1.65	1.70	34
Faizabad	22.6	92	8.1	1.53	1.74	-
Hardinath	15.8	92	6.6	1.54	1.88	30
Hazaribag	21.9	426	6.7	1.58	1.72	76
IIRR	70.0	327	8.3	1.63	1.72	78
Nepalgunj	12.2	85	7.1	1.43	1.78	32
Paramakudi	14.2	270	7.8	-	-	-
Patna	25.6	161	7.5	1.67	1.42	36
Raipur	10.6	173	7.1	1.74	1.72	30
Rajshahi	33.3	70	7.1	1.73	1.64	78
Rewa	4.8	200	7.7	1.58	1.52	78
Sabour	4.3	93	7.8	1.61	1.61	78
Tarahara	6.0	100	6.9	1.27	1.47	30
Tripura	3.7	122	4.6	1.73	1.75	48
Varanasi	8.0	74	8.4	1.72	1.69	34

**Fig. 3:**
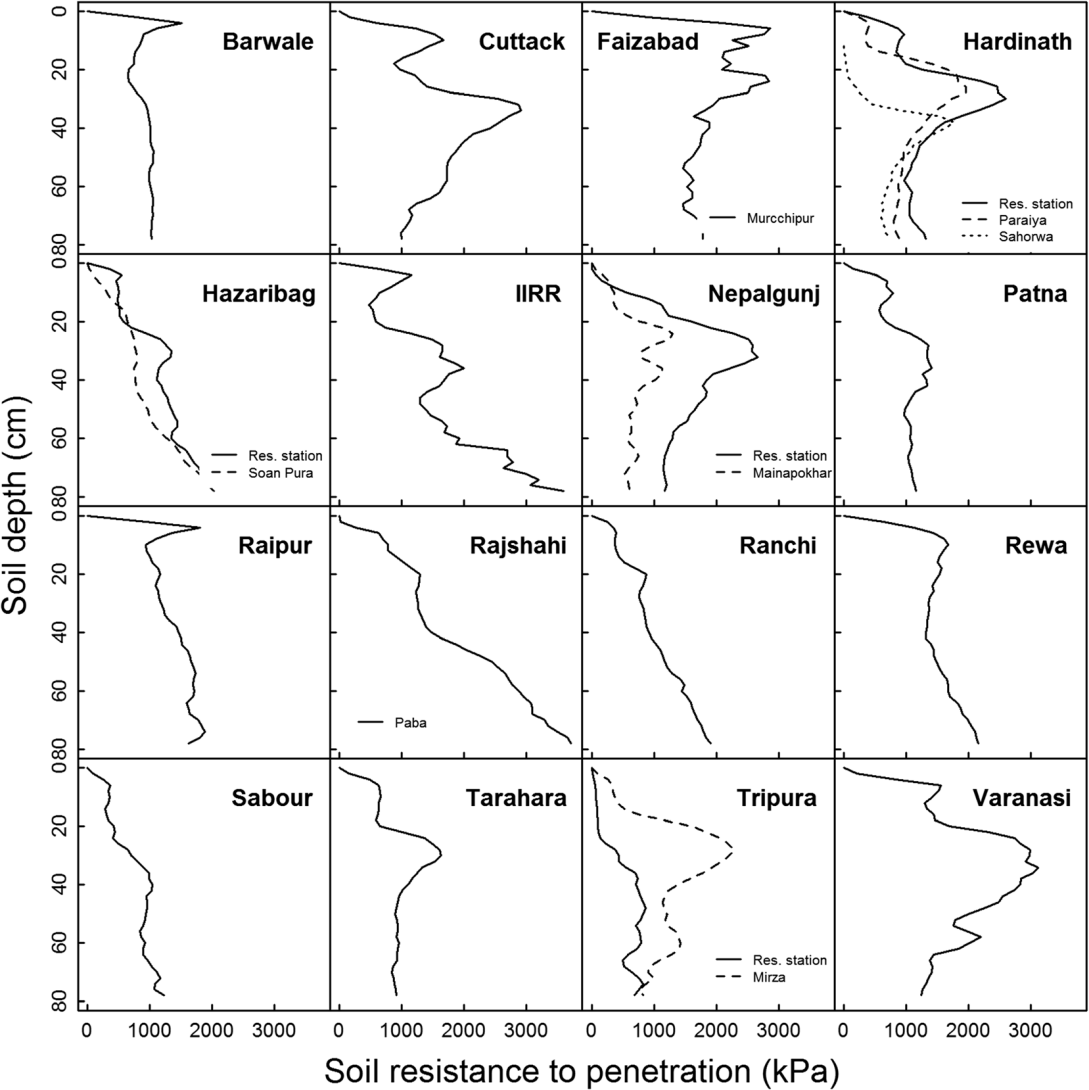
Soil resistance to penetration with depth as measured by cone penetrometer. Measurements were conducted at research station sites, unless otherwise indicated by the name of the on-farm site. The volumetric water content of the surface soil at the time of penetrometer reading is shown in Supp. Table 2

Penetrometer measurements indicated distinct hardpans at the three sites in Nepal (Hardinath, Nepalgunj, and Tarahara) as well as at four sites in India (Cuttack, Faizabad, Tripura on-farm, and Varanasi) where the maximum resistance to penetration was near the depth of 30 cm (Fig. 3). Minor hardpans were observed at Faizabad and Patna. In contrast, the soil resistance to penetration progressively increased with depth - indicating no apparent hardpan - at Barwale, Hazaribag, IIRR, Ranchi, Raipur, Rajshahi, Rewa, Sabour, and Tripura on-station (Fig. 3). These differences in presence of hardpans among sites appeared to be independent of the surface volumetric soil moisture content at the time of penetrometer measurements, which ranged from 17% to fully flooded (Supp. Table 2).

Compared to nearby on-farm sites, the research station sites showed significantly higher exchangeable K, bulk density at 25–30 cm, maximum penetrometer readings, and depth of the maximum penetrometer readings (Table 3; Supp. Table 4).

**Table 3:** Comparison of soil-related parameters between research stations and on-farm sites by t-test. For each parameter, 5–6 research station sites were compared with 6–7 farm sites. Except for bulk density at 5–10 cm, % clay, and % silt, all other parameters measured showed greater average values at the research station sites than at the on-farm sites

	Mean values			
	On-farm	Research station	t-value	*P*-value
Avail-P (ppm)	12.7	13.5	−0.1	0.92
Exch-K (ppm)	81.0	170	3.38	0.01*
pH	6.00	6.81	−1.02	0.33
bd 5–10 cm (g cm-3)	1.61	1.57	0.73	0.48
bd 25–30 cm (g cm-3)	1.56	1.73	−2.93	0.02*
%Clay	29.7	22.0	1.37	0.2
%Silt	41.3	37.6	0.59	0.57
%Sand	29.0	40.4	−1.56	0.15
Max penetrom reading (kPa)	2057	2066	9.57	<0.001***
Depth of max penetrom reading (cm)	33.3	52.8	3.37	0.02*
Penetrom reading at 30 cm (kPa)	1320	1524	−0.37	0.72

Soil water retention at 300 kPa and 500 kPa was highly correlated with clay content (Supp. Table 3), and was greatest in soil from both depths sampled at Barwale, Coimbatore, and Hazaribag (Supp. Fig. 1), which were the sites with the highest clay content (Fig. 2). The sites with least water retention were different at the two depths sampled; soil from Faizabad, IIRR, and Varanasi showed least water retention at 5–10 cm, whereas soil from Nepalgunj, Raipur, and Varanasi showed least water retention at 25–30 cm (Supp. Fig. 1), with Varanasi notably having the highest sand content (Fig. 2).

### Environmental characterization of the drought stress treatments

**Fig. 4:**
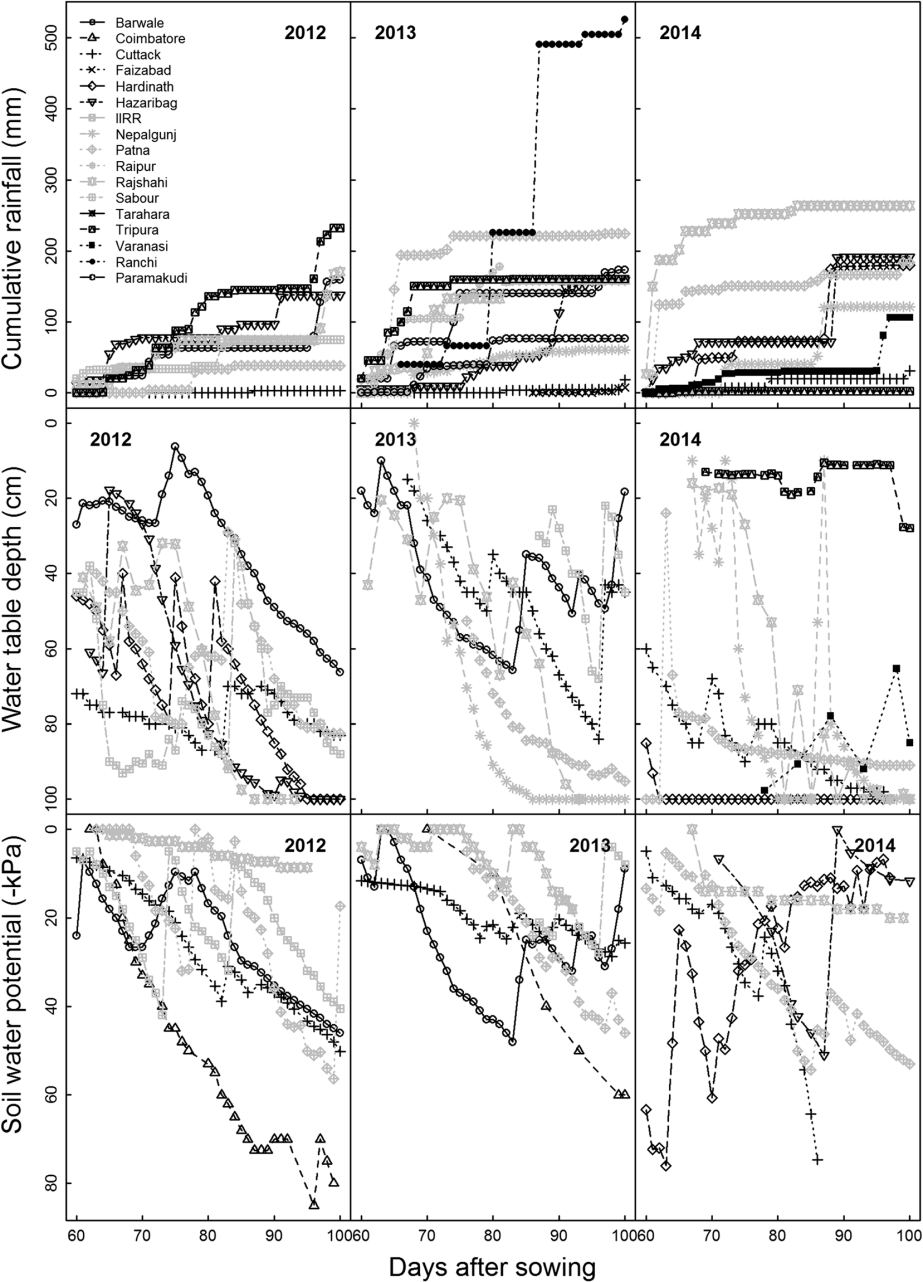
Rainfall, water table depth, and soil water potential readings at a depth of 30 cm measured from 60 to 100 days after sowing at research station sites during the rice drought screening trials in 2012, 2013, and 2014

Rainfall from 60 to 100 DAS ranged across sites and years from 2 mm in Tripura 2014 to 526 mm in Ranchi in 2013 (Fig. 4; Supp. Table 5). On average, 2012 was the lowest rainfall year with 96 mm across sites and 2014 the highest with 175 mm across sites, followed by 2013 with 165 mm. The distribution of rainfall within the 60–100 DAS period also varied considerably among sites (Fig. 4).

Soil water potential and water table depth also ranged greatly across sites and years, and fluctuated over the course of each trial (Fig. 4). The water table at several sites in each year reached the maximum observable depth of 1 m. The minimum soil water potential at a depth of 30 cm was observed in Coimbatore in 2012 and 2013, and in Hardinath in 2014. In general, lower rainfall resulted in deeper water table depths and more negative soil water potentials. However, some exceptions were observed - such as in the Tripura 2014 trial in which the water table depth remained shallow despite experiencing low rainfall.

### Yield reduction by drought

The yield reduction ranged from 7% (Hardinath) to 89% (IIRR) in 2012, from 12% (Tripura) to 88% (Coimbatore) in 2013, and from −5% (Nepalgunj) to 85% (Faizabad) in 2014 (Table 4). In 2012, two sites (Coimbatore and Hazaribag) faced severe stress and five sites (Barwale, Nepalgunj, Patna, Rewa, and Tripura) experienced moderate stress. In 2013, Coimbatore and Hazaribag encountered extreme and severe stress, respectively, and five sites (IIRR, Nepalgunj, Patna, Raipur, and Ranchi) experienced moderate stress. The greatest % yield reduction on average was observed in 2014 with Ranchi reporting extreme stress and five sites (Cuttack, Faizabad, Hardinath, IIRR, and Tripura) experiencing severe stress. Across the three years of this study, Hazaribag achieved the most consistent stress levels with yield reductions of 50% or more.

**Table 4:** Average yield (t ha^−1^) in the irrigated control and drought treatments and their corresponding yield reduction (%) from drought screening trials at research station sites from 2012 to 2014. The severity of drought stress at each site was classified based on yield reduction compared to the irrigated control as described by Kumar et al. (2009)

	2012				2013				2014			
Location	Yield control	Yield stress	% yield reduction	Stress level	Yield control	Yield stress	% yield reduction	Stress level	Yield control	Yield stress	% yield reduction	Stress level
Barwale	6.04	3.54	41.39	Moderate	5.63	4.04	28.20	Mild	-	-	-	-
Coimbatore	4.61	1.13	75.48	Severe	4.32	0.53	87.77	Extreme	-	-	-	-
Cuttack	-	0.62	-	-	-	1.88	-	-	2.56	0.52	79.53	Severe
Faizabad	4.45	3.61	18.81	Non stress	5.06	3.78	25.25	Mild	4.09	0.60	85.27	Severe
Hardinath	4.59	4.27	7.03	Non stress	3.53	2.84	19.50	Non stress	3.87	1.12	71.08	Severe
Hazaribag	5.25	1.81	65.46	Severe	4.80	1.54	68.03	Severe	6.00	2.95	50.80	Moderate
IIRR	4.62	0.51	88.99	Extreme	5.45	3.24	40.53	Moderate	5.65	0.96	83.03	Severe
Nepalgunj	3.14	2.12	32.57	Moderate	4.15	2.47	40.42	Moderate	4.09	4.31	−5.21	Non stress
Paramakudi	-	1.13	-	-	-	0.35	-	-	-	-	-	-
Patna	5.92	2.99	49.51	Moderate	5.75	3.46	39.86	Moderate	8.36	5.11	38.82	Moderate
Raipur	6.18	4.49	27.37	Mild	5.15	3.29	36.05	Moderate	6.38	2.46	61.53	Moderate
Rajshahi	4.10	3.77	8.10	Non stress	3.63	2.97	18.15	Non stress	3.68	3.37	8.22	Non stress
Ranchi	6.07	4.41	27.48	Mild	3.92	1.85	52.83	Moderate	2.52	0.09	96.55	Extreme
Rewa	2.71	1.71	36.80	Moderate	-	4.45	-		-	-	-	-
Sabour	5.14	3.65	28.86	Mild	-	1.45	-	-	4.58	4.30	6.01	Non stress
Tarahara	4.21	3.06	27.23	Mild	4.72	4.09	13.34	Non stress	-	-	-	
Tripura	5.62	3.51	37.46	Moderate	5.80	5.08	12.47	Non stress	5.90	1.69	71.40	Severe
Varanasi	-	-	-	-	-	-	-	-	8.28	7.81	5.75	Non stress

### Relating soil and environmental parameters to yield reduction by drought

In order to determine the best model for yield reduction under reproductive stage drought, stepwise multiple linear regression analysis was conducted using % yield reduction as the dependent variable and rainfall and soil parameters (bulk density 5–10 cm and 25–30 cm, pH, available P, % clay, % silt, % sand, water retention at 10 kPa from 5 to 10 cm, maximum penetrometer reading, depth of the maximum penetrometer reading, and penetrometer reading at 30 cm) as independent variables (Tables 4 and 5). In the fitted model, minimum soil water potential, depth of maximum penetrometer reading, and penetrometer reading at 30 cm showed positive correlations with % yield reduction, and % silt showed a negative relationship.

**Table 5:** Yield reduction by drought across three years of study as a function of rainfall and soil-related parameters as determined by step-wise multiple regression

Predictor	Coefficient	*P*-value
Intercept	−11.77	0.4858
Min. SWP 60–100 DAS	0.7	<0.001
% Silt	−0.35	0.0667
Depth of max penetrom reading	0.38	0.009
Penetrom reading at 30 cm	0.01	0.0328

*P*-value of fitted model < 0.001

r^2^ of fitted model 0.6085

To further group the rainfall and soil parameters measured with % yield reduction by drought across sites, a principal component analysis (PCA) was conducted (Table 6). Following Kaiser’s stopping rule and Scree test, the PCA yielded four principal components explaining a total of 66.4% of the variance for the entire set of variables collected from 16 different locations. The first four principal components (PC1, PC2, PC3 and PC4) showed eigenvector values >2.00 (PC4 = 2.1846).

**Table 6:**
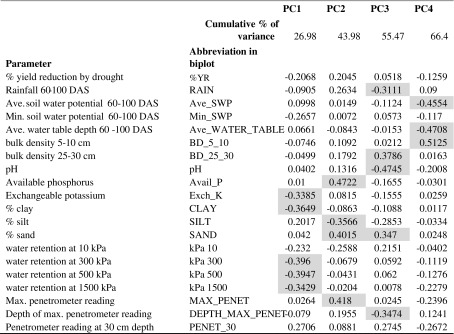
Eigenvector values from a principal component analysis of yield reduction by drought a various soil characteristics at the research station drought screening sites characterized in this study.

Shaded values are the highest factor loading values for each principal component

Parameters with factor loading values greater than 0.3 were identified from each principal component (Table 6). The first principal component (PC1) accounted for 27% of the total variation in the data, with water retention at 500 kPa, 300 kPa, and 1500 kPa, % clay, and exchangeable K^+^ contributing most to the variation. The second principal component (PC2) contributed 17% of the total variation, with % sand, % silt, available P, and the maximum penetrometer reading showing highest factor loading values. The third principal component accounted for 11.5% of the total variation in which % sand, pH, bulk density at 25–30 cm, depth of the maximum penetrometer reading, and rainfall contributed most to the variation. The fourth principal component contributed only 10.9% to the variation, which was most related to bulk density at 5–10 cm, average soil water potential, and average water table depth. Among study sites, Barwale, Coimbatore, Faizabad, and Hazaribag showed the strongest effect on PC1, IIRR on PC2, and Cuttack, Sabour, and Tripura on PC3 (Supp. Table 6).

**Fig. 5:**
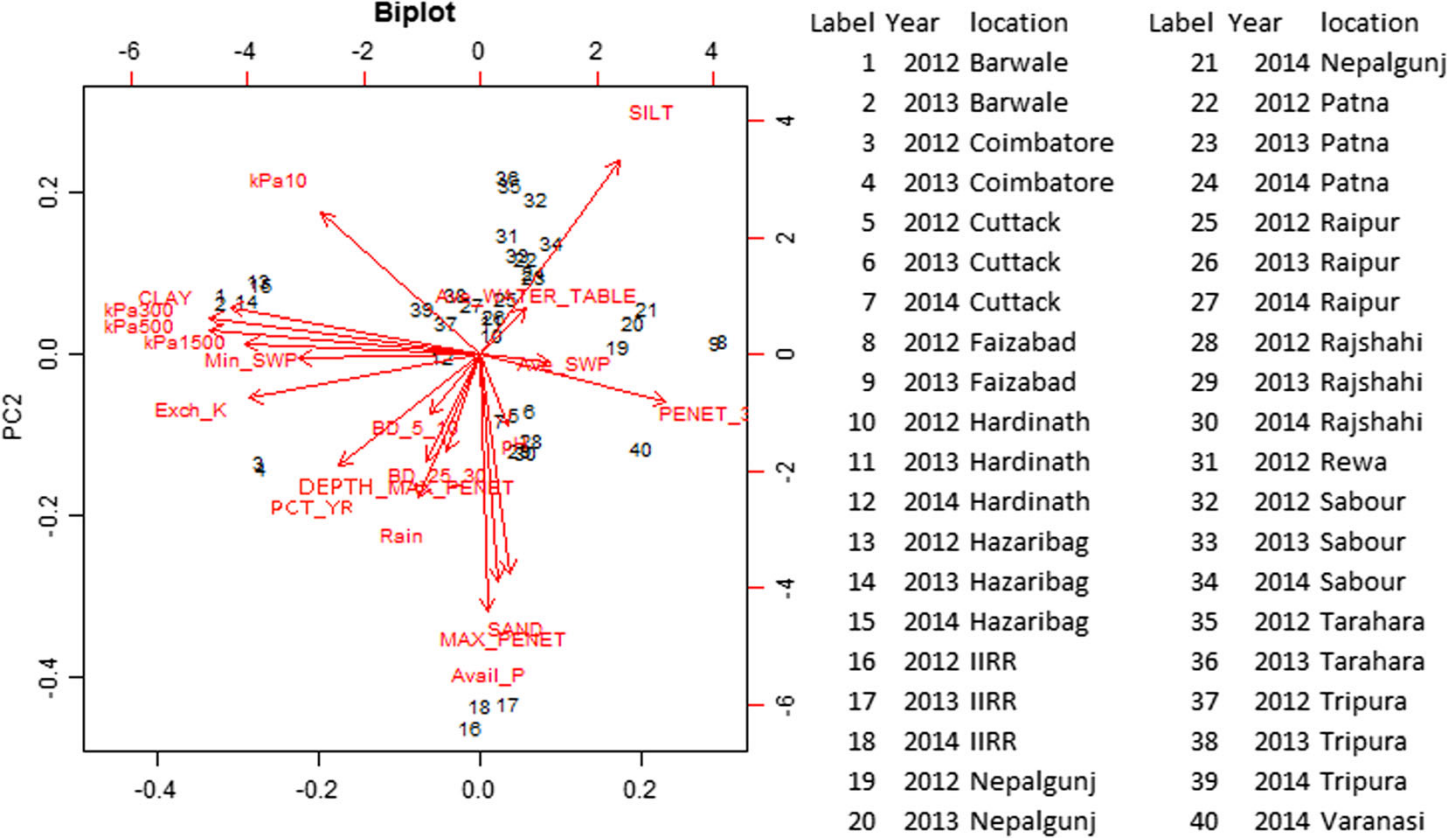
Biplot from a principal component analysis of yield reduction by drought a various soil characteristics at the research station drought screening sites characterized in this study. Parameter abbreviations are described in Table 5

A biplot of PC1 and PC2 was constructed to visualize the groupings of factors and locations, and to effectively interpret their load on the principal components (Fig. 5). The % yield reduction by drought grouped with rainfall, depth of maximum penetrometer reading, and bulk density at 5–10 cm and 25–30 cm. Site IIRR grouped with % sand, whereas Barwale and Hazaribag grouped with % clay.

## Discussion

This study involved characterization of the soil properties and drought stress severity at 18 research stations involved in selection of high-yielding, drought tolerant rice breeding lines in drought-prone regions of Bangladesh, India, and Nepal. The analysis of the relationships between environmental characteristics and yield reduction by drought at rice varietal screening sites has revealed some unexpected trends: 1) the absence of a distinct soil hardpan at many sites, and 2) the grouping of depth of maximum soil resistance to penetration with yield reduction by drought, and 3) a lack of direct relationship of yield reduction by drought with soil clay content. Such understanding of the target environment for crop improvement may be useful for extrapolating the varietal screening results to other drought-prone rice-growing regions and subsequent recommendation of varieties.

Rainfed lowland rice-growing areas are well-documented to show a high degree of variability among locations (Wade et al. 1999), and this variability was further reflected here by the range of soil types, nutrient levels, and physical properties among sites. One feature thought to be typical to most rainfed lowland fields is the presence of a soil hardpan due to the practice of soil puddling, which was notably absent in many of the sites characterized in this study. Hardpans that are detectable by penetrometer profiles have been characterized in lowland rice fields in Bangladesh (Samson et al. 2002) and Laos (Vial et al. 2013), and interestingly these studies and a larger survey across rice-growing areas of South and Southeast Asia (Cairns et al. 2011) have reported greater maximum penetrometer readings (ranging up to 8.8 MPa) than those in this study (up to 3.7 MPa only).

In addition to innate soil properties, the absence of a hardpan may be related to the frequent use of dry direct seeding as an establishment practice at some sites, as is the case in Paramakudi. With puddling and transplanting not being a regular practice season after season, a detectable hard pan may not have developed; this may have been the case in the relatively newly-cultivated on-station sites including Barwale, Patna, and Tripura (Table 1). The similarities between soil resistance to penetration profiles at research station sites with on-farm sites (Fig. 3) suggests that these soil physical observations represent the area of the experimental fields and are not specific to research station sites. Another reason for the frequent absence of a detectable hardpan and lower penetrometer readings in this study may be due to the focus on drought screening sites that were actively managed to be drained during the crop reproductive stage. Such soil drying often leads to soil cracking (depending on the clay content and presence of swelling-shrinking clay minerals), which may have effectively disrupted the hardpan at these sites. Vial et al. (2013) observed the maximum penetrometer reading in rice soils of central Lao PDR to be reduced from 3.1–5.0 MPa to 2.5–3.2 MPa by mechanical disruption of hardpans. Indeed, anecdotal observations of soil cracking have been much more frequent at sites in the current study such as Barwale, Hazaribag, and Patna (in which hardpans were not detected by penetrometer) compared to Cuttack, Hardinath, Nepalgunj, and Tarahara which typically show much less soil cracking and where hardpans were detected. Due to the detrimental effects of puddling on soil physical properties including increased bulk density and crack formation, a separate study conducted at Patna recently suggested omission of puddling for rice cultivation in that soil (Mondal et al. 2016).

Soil cracking may have different effects on the crop during drought stress: larger cracks may allow better infiltration of rainfall and rewetting by bypass flow, but large cracks also provide more surface area for evaporation and can increase lateral water loss (Tuong et al. 1996). In the context of this study, the soil cracks also presented difficulties in obtaining reliable tensiometer readings, especially under severe drought stress - although the practice of installing the tensiometers at a depth of 30 cm may reduce interruptions in the contact between the soil and the porous cup of the instrument, as well as the likelihood of tensiometer failure at high soil water tensions. Intermittent rainfall may also provide conditions more favorable for tensiometer use; Dasgupta et al. (2015) reported high correlations between tensiometer readings at 10 cm depth with modeled soil water potential values in rice fields up to tensions of 90 kPa under intermittent drought stress in rice soils of West Bengal, India.

High bulk density values ranging up to 1.88 g cm^−3^ were another notable feature of the soils in this study, but were not correlated with soil resistance to penetration. Although high bulk density and soil resistance to penetration have been attributed to reduced root growth due to mechanical impedance (Bengough and Mullins 1990), there were likely genotype-specific responses to these conditions in the drought screening trials. In experiments with combined soil compaction and soil drying treatments, Hoque and Kobata (2000) reported decreased biomass, yield, and yield components; but Kobata et al. (2000) observed that some drought resistant genotypes maintained relatively better root growth across treatments. Furthermore, Samson et al. (2002) reported that some rice varieties could continue increasing their root length density even during the crop reproductive stage as the soil strength increased during drought stress, whereas the root growth of other genotypes was impeded. In a previous study characterizing drought breeding lines at some of the sites characterized in the current study, root length density values were generally lower at Raipur and Hazaribag where bulk density ranged up to 1.74 g cm^−3^ than at the International Rice Research Institute where bulk density values were about 1.0 g cm^−3^ (Anantha et al. 2016). Based on these root growth responses, site variation in bulk density may affect the ability to discriminate among genotypes under drought stress. The grouping of % yield reduction with bulk density in the PCA (Fig. 5) further points to the importance of bulk density in rice response to drought in this region.

The correlation of % yield reduction by drought with the absence of a hardpan (i.e. with deeper maximum penetrometer readings depths) was unexpected based on previous reports of factors affecting rainfed lowland rice yield under drought. Another unexpected trend observed in this study was the lack of relationship between yield reduction by drought and soil clay content. Soil clay content was expected to be related to water retention as it is typically highlighted in rice rainfed lowland site comparisons (e.g. Wade et al. 1999; Inthavong et al. 2012), but only a negative relationship between silt content and yield reduction by drought was observed in the PCA and multiple regression. This negative relationship with silt may reflect that yield reduction by drought was related to a high clay content in some cases and a high sand content in other cases; of the sites with the most consistently high % yield reduction by drought, two (Hazaribag and Coimbatore) were among the highest-clay sites, and one (IIRR) was one of the highest-sand sites.

Rainfall was clearly a key factor in yield reduction by drought based correlations and groupings in the principal component analysis (Fig. 5, Supp. Table 3). At Nepalgunj in 2014, a single rainfall event of 69 mm at 87 DAS resulted in similar mean yield in the drought stress treatment as in the irrigated control. However, the sites with lowest rainfall did not always show the greatest yield reduction by drought due to other site characteristics. Topography has been reported to be an explanatory factor of rice yield in drought-prone rainfed lowlands in Lao PDR (Inthavong et al. 2011; Inthavong et al. 2012); this large range in altitudes and resulting variability in drainage potential among rainfed rice fields is more typical of Southeast Asia. The current study was conducted in regions including the barind tract areas of Bangladesh, Indo-gangetic plain of India, and the *terai* of Nepal, all of which show relatively little variation in topography. Some exceptions were Tripura, which is in a region characterized as *lunga* land featuring rolling hills and where the low-lying topography of the drought screening field resulted in shallow water tables despite low rainfall, and Sabour, which is located near the Ganges River and despite low rainfall experienced severe flooding in 2013 from river water that caused the loss of the irrigated control trial.

The delayed planting of the drought stress treatment compared to the irrigated control may have been a factor affecting % yield reduction in addition to drought at some sites. Some rice genotypes appear to have an optimal sowing date after which plant growth, flowering time, and grain yield may be affected (Sarkar and Reddy 2006; Singh et al. 2015). However, in the current study, the genotypes evaluated did not appear to be photoperiod sensitive nor did the trials run late enough to experience unfavorable temperatures.

Another parameter typically associated with rainfed rice production in Southeast Asia is low-fertility soil (Fukai and Ouk 2012), but this did not appear to be a predominant factor based on the large range of soil P and K values observed across sites (Table 2). Soil organic matter has been reported to be quite stable across seasons in flooded lowland rice fields (Pampolino et al. 2008); although soil organic matter may be variable among drought-prone rice fields and may affect soil water retention, it was not quantified here.

The main difference between research station and on-farm sites was in terms of soil compaction. This was also observed by Cairns et al. (2011) and is likely due to predominance of mechanical soil preparation at research stations and manual or animal soil preparation at on-farm sites. The similarities between the research station and on-farm sites suggests that the detailed soil characterization presented here can be scaled up to better understand the relationships between soil characteristics and yield reduction by drought in the region. The environmental characterization in this study could potentially be used for recommendation and dissemination of drought-tolerant varieties. By analyzing and characterizing the accumulated soil related data in government department or agricultural universities in India, Bangladesh and Nepal, one can identify which area of the province or state will be more prone to drought under different ranges of deficit rainfall, and to prioritize dissemination of drought tolerant varieties.

This study provides detailed information about the drought stress severity in target drought-prone rice growing areas of South Asia, but it should be noted that the drought screening trials in this study were managed to target reproductive stage; therefore, rice fields in the same regions that are under completely rainfed conditions likely experience different soil water potential and water table depth values. Other types of drought stress that occur at seedling and vegetative stage also present significant challenges to rainfed rice farmers, and may result in different relationships between crop performance and soil characteristics than those observed here. Although characterizing the types and severities of drought stress that occur in rainfed farmers’ fields will require additional investigation, this study of managed research station trials provides a starting point for understanding the target environments for which drought tolerant rice varieties are being developed that can be further scaled up coupled with modeling and remote sensing.

## Electronic supplementary material

ESM 1 (DOCX 100 kb)
