# Supplementary material for: Depth of soil compaction predominantly affects rice yield reduction by reproductive-stage drought at varietal screening sites in Bangladesh, India, and Nepal
Source: Plant Soil. 2017 May 10;417(1):377–92. doi: 10.1007/s11104-017-3265-2 (PMC6560918; doi:10.1007/s11104-017-3265-2)
Supplement: Supplementary file 1 — (DOCX 100 kb) [file 11104_2017_3265_MOESM1_ESM.docx]

**Supplemental Material**

Singh et al. Depth of soil compaction predominantly affects rice yield reduction by reproductive-stage drought at varietal screening sites in Bangladesh, India, and Nepal

Supp. Table 1. The range and average number of days to flowering (DTF) in the irrigated control and drought treatments at research station sites from 2012 - 2014.

|  | **2012** | | | | **2013** | | | | **2014** | | | |
| --- | --- | --- | --- | --- | --- | --- | --- | --- | --- | --- | --- | --- |
|  | **Control** | | **Stress** | | **Control** | | **Stress** | | **Control** | | **Stress** | |
| Location | DTF range | Ave. DTF | DTF range | Ave. DTF | DTF range | Ave. DTF | DTF range | Ave. DTF | DTF range | Ave. DTF | DTF range | Ave. DTF |
| Barwale | 90-104 | 99 | 83-96 | 90 | 83-92 | 88 | 81-93 | 88 | - | - | - | - |
| Coimbatore | 81-95 | 89 | 82-95 | 91 | 80-92 | 87 | 81-99 | 90 | - | - | - | - |
| Cuttack | - | - | 68-118 | 87 | - | - | 83-95 | 91 | 67-79 | 74 | 80-86 | 84 |
| Faizabad | 91-106 | 98 | 79-92 | 86 | 76-97 | 87 | 72-87 | 80 | 81-96 | 88 | 79-96 | 88 |
| Hardinath | 74-119 | 91 | 73-117 | 86 | 78-118 | 92 | 60-114 | 72 | 86-108 | 100 | 78-93 | 87 |
| Hazaribag | 84-101 | 92 | 86-107 | 98 | 88-103 | 97 | 83-92 | 89 | 84-94 | 89 | 87-97 | 92 |
| IIRR | 90-104 | 95 | 90-108 | 98 | 90-107 | 100 | 85-99 | 92 | 97-113 | 106 | 100-116 | 106 |
| Nepalgunj | 81-122 | 93 | 77-128 | 89 | 83-115 | 93 | 77-108 | 87 | 76-100 | 88 | 75-98 | 88 |
| Paramakudi | - | - | 61-103 | 83 | - | - | 70-84 | 77 | - | - | - | - |
| Patna | 72-104 | 84 | 73-92 | 81 | 70-87 | 79 | 70-85 | 78 | 76-91 | 83 | 76-90 | 81 |
| Raipur | 86-104 | 96 | 89-99 | 95 | 67-82 | 76 | 67-82 | 76 | 81-97 | 87 | 80-95 | 87 |
| Rajshahi | 70-100 | 81 | 70-98 | 82 | 76-87 | 84 | 72-85 | 81 | 83-103 | 88 | 79-103 | 86 |
| Ranchi | 95-104 | 100 | 91-109 | 97 | 101-112 | 107 | 79-90 | 83 | - | - | - | - |
| Rewa | 73-99 | 86 | 74-94 | 84 |  |  |  |  |  |  |  |  |
| Sabour | 75-90 | 83 | 73-93 | 85 | 96-114 | 104 |  |  | 74-88 | 80 | 75-86 | 81 |
| Tarahara | 69-110 | 89 | 88-123 | 97 | 76-118 | 90 | 77-111 | 88 |  |  |  |  |
| Tripura |  |  |  |  | 87-105 | 95 | 92-106 | 101 |  |  | 76-88 | 81 |
| Varanasi | - | - | - | - | - | - | - | - | 75-97 | 87 | 77-87 | 81 |

Supp. Table 2. Soil volumetric water content (θ_v_) from 0-5 cm at the time of penetrometer readings.

|  |  | θ_v_ 0-5 cm at time of penetrometer reading |
| --- | --- | --- |
| **Barwale** | Res. station | 39.4 |
| **Cuttack** | Res. station | 24.6 |
| **Faizabad** | Murcchipur | 17.9 |
| **Hardinath** | Paraiya, Siraha | 38.3 |
|  | Res. station | 31.0 |
|  | Sahorwa | recently flooded |
| **Hazaribag** | Res. station | 38.2 |
|  | Soan Pura, Chotra | 40.4 |
| **IIRR** | Res. station | 29.5 |
| **Nepalgunj** | Mainapokhar | 35.0 |
|  | Res. station | 14.2 |
| **Patna** | Res. station | 38.3 |
| **Raipur** | Res. station | 34.4 |
| **Rajshahi** | Paba, Rajshahi | 16.8 |
| **Ranchi** | Res. station | 36.0 |
| **Rewa** | Res. station | 35.4 |
| **Sabour** | Res. station | 37.0 |
| **Tarahara** | Res. station | 40.9 |
| **Tripura** | Mirza | 25.7 |
|  | Res. station | 42.6 |
| **Varanasi** | Res. station | 34.3 |

Supp. Table 3. Correlation matrix generated as part of the Principle Component Analysis relating yield reduction by drought to rainfall and soil parameters across research station sites. YR: percent yield reduction by drought, RF: rainfall from 60-100 days after sowing, BD: bulk density, kPa: water holding capacity at a given pressure, MAX_PENET: maximum penetrometer reading, PENET_30: pentrometer reading at the depth of 30 cm.

| Traits | | PCT_  YR | | RAIN | | AVE_SWP | | Min_SWP | | AVE_  WATER_TABLE | | BD_  5_10_cm | | BD_  25_30_cm | | pH | Avail_P | | Exch-K | | CLAY | | SILT | | SAND | | kPA  10 | | kPA  300 | | kPa  500 | | kPa  1500 | | MAX_  PEN | DEPTH_MAX_PEN | |
| --- | --- | --- | --- | --- | --- | --- | --- | --- | --- | --- | --- | --- | --- | --- | --- | --- | --- | --- | --- | --- | --- | --- | --- | --- | --- | --- | --- | --- | --- | --- | --- | --- | --- | --- | --- | --- | --- |
| RAIN | 0.04 | |  | |  | |  | |  | |  | |  | |  | |  |  | |  | |  | |  | |  | |  | |  | |  | |  | |  |  |
| AVE_SWP | 0.09 | | 0.01 | |  | |  | |  | |  | |  | |  | |  |  | |  | |  | |  | |  | |  | |  | |  | |  | |  |  |
| Min_SWP | 0.69 | | -0.05 | | 0.17 | |  | |  | |  | |  | |  | |  |  | |  | |  | |  | |  | |  | |  | |  | |  | |  |  |
| AVE_WATER_TABLE | -0.10 | | -0.07 | | 0.24 | | -0.09 | |  | |  | |  | |  | |  |  | |  | |  | |  | |  | |  | |  | |  | |  | |  |  |
| BD_5_10_cm | 0.05 | | 0.16 | | -0.50 | | 0.05 | | -0.47 | |  | |  | |  | |  |  | |  | |  | |  | |  | |  | |  | |  | |  | |  |  |
| BD_25_30_cm | 0.13 | | 0.16 | | -0.11 | | -0.07 | | -0.19 | | 0.01 | |  | |  | |  |  | |  | |  | |  | |  | |  | |  | |  | |  | |  |  |
| pH | -0.09 | | 0.36 | | 0.34 | | -0.25 | | 0.11 | | -0.14 | | -0.22 | |  | |  |  | |  | |  | |  | |  | |  | |  | |  | |  | |  |  |
| Avail_P | 0.36 | | 0.44 | | 0.04 | | 0.06 | | -0.12 | | 0.11 | | 0.04 | | 0.33 | |  |  | |  | |  | |  | |  | |  | |  | |  | |  | |  |  |
| Exch-K | 0.35 | | 0.31 | | -0.08 | | 0.39 | | -0.32 | | 0.19 | | 0.05 | | 0.32 | | 0.22 |  | |  | |  | |  | |  | |  | |  | |  | |  | |  |  |
| CLAY | 0.24 | | 0.22 | | -0.16 | | 0.33 | | -0.09 | | 0.21 | | 0.06 | | 0.09 | | -0.21 | 0.68 | |  | |  | |  | |  | |  | |  | |  | |  | |  |  |
| SILT | -0.37 | | -0.25 | | 0.15 | | -0.24 | | 0.15 | | -0.26 | | -0.42 | | 0.17 | | -0.37 | -0.38 | | -0.29 | |  | |  | |  | |  | |  | |  | |  | |  |  |
| SAND | 0.20 | | 0.10 | | -0.04 | | 0.01 | | -0.09 | | 0.12 | | 0.37 | | -0.22 | | 0.49 | -0.07 | | -0.37 | | -0.78 | |  | |  | |  | |  | |  | |  | |  |  |
| kPA10 | 0.03 | | -0.12 | | -0.36 | | 0.16 | | 0.14 | | -0.21 | | 0.30 | | -0.39 | | -0.50 | 0.28 | | 0.41 | | -0.02 | | -0.25 | |  | |  | |  | |  | |  | |  |  |
| kPA300 | 0.27 | | 0.07 | | -0.11 | | 0.49 | | -0.03 | | 0.08 | | 0.08 | | -0.02 | | -0.15 | 0.75 | | 0.84 | | -0.43 | | -0.13 | | 0.53 | |  | |  | |  | |  | |  |  |
| kPa500 | 0.30 | | 0.10 | | -0.09 | | 0.51 | | -0.02 | | 0.09 | | 0.13 | | -0.03 | | -0.09 | 0.72 | | 0.83 | | -0.43 | | -0.13 | | 0.51 | | 0.99 | |  | |  | |  | |  |  |
| kPa1500 | 0.45 | | 0.04 | | -0.15 | | 0.55 | | 0.22 | | -0.19 | | -0.01 | | -0.14 | | -0.01 | 0.43 | | 0.57 | | -0.29 | | -0.09 | | 0.54 | | 0.73 | | 0.75 | |  | |  | |  |  |
| MAX_PENET | 0.18 | | 0.23 | | 0.07 | | -0.17 | | 0.21 | | -0.07 | | 0.32 | | 0.30 | | 0.65 | -0.05 | | -0.10 | | -0.44 | | 0.49 | | -0.28 | | -0.06 | | 0.00 | | 0.14 | |  | |  |  |
| DEPTH_MAX_PENET | 0.23 | | 0.46 | | -0.29 | | 0.04 | | -0.04 | | 0.02 | | -0.07 | | 0.17 | | 0.41 | 0.08 | | 0.11 | | -0.04 | | -0.04 | | -0.02 | | -0.12 | | -0.10 | | 0.32 | | 0.32 | |  |  |
| PENET_30_  CM | -0.17 | | -0.31 | | 0.29 | | -0.36 | | 0.28 | | -0.23 | | 0.29 | | 0.08 | | 0.09 | -0.47 | | -0.53 | | 0.09 | | 0.26 | | -0.29 | | -0.42 | | -0.39 | | -0.46 | | 0.38 | | -0.53 |  |

Supp. Table 4. Soil available P, exchangeable K, and pH from 0-15 cm, and bulk density (bd) from 5-10 cm and 25-30 cm at on-farm sites characterized in this study.

| **On-farm site** | **Nearby research station** | **Avail-P (mg kg^-1^)** | **Exch-K (mg kg^-1^)** | **pH** | **bd 5-10 cm**  **(g cm^-3^)** | **bd 25-30 cm**  **(g cm^-3^)** | **%Clay** | **%Silt** | **%Sand** |
| --- | --- | --- | --- | --- | --- | --- | --- | --- | --- |
| Murchhipur | Faizabad | 53.4 | 52 | 7.8 | 1.63 | 1.58 | 17.4 | 47 | 35.6 |
| Baluwa | Hardinath | 8.1 | 40 | 4.5 | 1.61 | 1.58 | 30.4 | 44 | 25.6 |
| Sahorwa | Hardinath | 9.2 | 24 | 7.8 | 1.61 | 1.49 | 22.4 | 52 | 25.6 |
| Chauria | Hazaribag | 4.3 | 196 | 4.5 | 1.52 | 1.68 | 38.4 | 36 | 25.6 |
| Puraina | Nepalgunj | 4.0 | 46 | 4.7 | 1.50 | 1.43 | 34.4 | 46 | 19.6 |
| Khokhum | Rewa | 5.1 | 143 | 7.5 | - | - | 44.4 | 42 | 13.6 |
| Mirza | Tripura | 5.00 | 67.2 | 5.18 | 1.76 | 1.58 | 20.5 | 22 | 57.5 |

Supp. Table 5. Summary of rainfall, soil water potential, and water table measurements shown in Fig. 5.

| **Year** | **location** | **Rainfall 60-100 DAS (mm)** | **Ave. soil water potential**  **60-100 DAS (-kPa)** | **Min. soil water potential**  **60-100 DAS (-kPa)** | **Ave. water table depth**  **60-100 DAS (cm)** | |  |
| --- | --- | --- | --- | --- | --- | --- | --- |
| 2012 | Barwale | 120 | 26 | 46 | | 33 | |
|  | Coimbatore | - | 50 | 85 | | - | |
|  | Cuttack | 3 | 24 | 50 | | 78 | |
|  | Hardinath | - | 9 | 11 | | 71 | |
|  | Hazaribag | 127 | - | - | | 72 | |
|  | Paramakudi | 17 | - | - | | - | |
|  | Patna | 38 | 22 | 56 | | 63 | |
|  | Raipur | 95 | - | - | | - | |
|  | Rajshahi | 160 | 5 | 9 | | 65 | |
|  | Rewa | - | 1 | 4 | | - | |
|  | Sabour | 75 | 21 | 42 | | 75 | |
|  | Tripura | 232 | - | - | | - | |
| 2013 | Barwale | 228 | 25 | 48 | | 42 | |
|  | Coimbatore | - | 37 | 60 | | - | |
|  | Cuttack | 18 | 20 | 29 | | 48 | |
|  | Faizabad | 41 | - | - | | - | |
|  | Hardinath | 127 | - | - | | - | |
|  | Hazaribag | 154 | - | - | | - | |
|  | IIRR | - | 21 | 26 | | - | |
|  | Nepalgunj | 61 | - | - | | 81 | |
|  | Paramakudi | 76 | - | - | | - | |
|  | Patna | 205 | 27 | 46 | | 82 | |
|  | Raipur | 178 | - | - | | - | |
|  | Rajshahi | 159 | 5 | 18 | | 49 | |
|  | Ranchi | 526 | - | - | | - | |
|  | Rewa | 248 | - | - | | - | |
|  | Sabour | 292 | 17 | 28 | | 39 | |
|  | Tripura | 141 | - | - | | - | |
| 2014 | Cuttack | 31 | 29 | 75 | | 85 | |
|  | Faizabad | 192 | - | - | | - | |
|  | Hardinath | 180 | 29 | 76 | | 99 | |
|  | Hazaribag | 192 | - | - | | - | |
|  | IIRR | - | 22 | 51 | | - | |
|  | Nepalgunj | 121 | - | - | | 74 | |
|  | Patna | 184 | 32 | 54 | | 86 | |
|  | Rajshahi | 237 | 15 | 20 | | 69 | |
|  | Sabour | 406 | - | - | | - | |
|  | Tripura | 2 | - | - | | 14 | |
|  | Varanasi | 106 | 11 | 12 | | 85 | |

Supp. Table 6. Loading values of each site/year in the principal component analysis of yield reduction by drought at various soil characteristics at the research station drought screening sites characterized

| Year | location | PC1 | PC2 | PC3 |
| --- | --- | --- | --- | --- |
| 2012 | Barwale | -4.7465 | 0.860681 | -0.26339 |
| 2013 | Barwale | -4.72517 | 0.740472 | 0.063851 |
| 2012 | Coimbatore | -4.05276 | -1.5393 | 0.61011 |
| 2013 | Coimbatore | -4.02061 | -1.63855 | 0.560334 |
| 2012 | Cuttack | 0.619091 | -0.86013 | -2.36586 |
| 2013 | Cuttack | 0.897743 | -0.80175 | -2.28201 |
| 2014 | Cuttack | 0.369691 | -0.94447 | -2.3111 |
| 2012 | Faizabad | 4.423379 | 0.217625 | 0.443709 |
| 2013 | Faizabad | 4.277608 | 0.184616 | 0.303778 |
| 2012 | Hardinath | 0.201165 | 0.282703 | -1.65708 |
| 2013 | Hardinath | 0.20153 | 0.494836 | -2.0078 |
| 2014 | Hardinath | -0.69519 | -0.02423 | -1.9167 |
| 2012 | Hazaribag | -4.03884 | 1.0584 | 0.40136 |
| 2013 | Hazaribag | -4.27304 | 0.769166 | 0.400357 |
| 2014 | Hazaribag | -4.02129 | 1.000592 | 0.57418 |
| 2012 | IIRR | -0.2032 | -5.37817 | 1.486459 |
| 2013 | IIRR | 0.509531 | -5.03614 | 1.476135 |
| 2014 | IIRR | -0.01612 | -5.08109 | 0.856271 |
| 2012 | Nepalgunj | 2.48865 | 0.124888 | -0.79991 |
| 2013 | Nepalgunj | 2.769039 | 0.450131 | -1.08764 |
| 2014 | Nepalgunj | 3.021745 | 0.657981 | -0.92263 |
| 2012 | Patna | 0.838339 | 1.372966 | 1.261766 |
| 2013 | Patna | 0.972741 | 1.12292 | 1.82087 |
| 2014 | Patna | 0.96125 | 1.187383 | 1.786703 |
| 2012 | Raipur | 0.454339 | 0.821859 | -0.12796 |
| 2013 | Raipur | 0.228617 | 0.564845 | 0.067647 |
| 2014 | Raipur | -0.169 | 0.710691 | -0.54643 |
| 2012 | Rajshahi | 0.921564 | -1.23468 | 0.727894 |
| 2013 | Rajshahi | 0.705439 | -1.37547 | 0.678529 |
| 2014 | Rajshahi | 0.825055 | -1.41702 | 0.996911 |
| 2012 | Rewa | 0.488901 | 1.721205 | 1.093255 |
| 2012 | Sabour | 1.012783 | 2.250962 | 2.229258 |
| 2013 | Sabour | 0.678374 | 1.453867 | 2.764047 |
| 2014 | Sabour | 1.295794 | 1.611889 | 3.363922 |
| 2012 | Tarahara | 0.535848 | 2.459413 | 0.25818 |
| 2013 | Tarahara | 0.488607 | 2.563426 | 0.253625 |
| 2012 | Tripura | -0.62528 | 0.476118 | -1.95061 |
| 2013 | Tripura | -0.4639 | 0.866889 | -2.17902 |
| 2014 | Tripura | -1.07381 | 0.660302 | -2.77353 |
| 2014 | Varanasi | 2.937876 | -1.35584 | -1.28749 |





Supp. Fig. 1. Soil water retention curves from research station sites, measured on loose soil samples using a pressure plate apparatus.
